# Simvastatin Enhances Stem Cell Osteogenesis and Reduces Peri-Implant Bone Loss: An In Vitro and a Randomized Clinical Study

**DOI:** 10.3390/ph19030368

**Published:** 2026-02-26

**Authors:** Asmaa Saleh, Shereen N. Raafat, Sherihan Ahmed Sayed, Mohamed Shamel, Sherif Shafik El Bahnasy, Sara F. El Shafei

**Affiliations:** 1 Department of Pharmaceutical Sciences, College of Pharmacy, Princess Nourah Bint Abdulrahman University, P.O. Box 84428, Riyadh 11671, Saudi Arabia; asali@pnu.edu.sa; 2 Department of Pharmacology, Faculty of Dentistry, The British University in Egypt, Al Sherouk City 11837, Cairo, Egypt; 3 Dental Science Research Group, Health Research Centre of Excellence, The British University in Egypt, Al Sherouk City 11837, Cairo, Egypt; 4 Department of Clinical Pharmacy and Pharmacy Practice, Faculty of Pharmacy, Galala University, New Galala City 43511, Suez, Egypt; sherihan.ahmed55@hotmail.com; 5 Department of Oral Biology, Faculty of Dentistry, The British University in Egypt, Al Sherouk City 11837, Cairo, Egypt; mohamed.shamel@bue.edu.eg; 6 Oral and Maxillofacial Radiology Department, Faculty of Dentistry, The British University in Egypt, Al Sherouk City 11837, Cairo, Egypt; sherif.shafik@bue.edu.eg; 7 Department of Removable Prosthodontics, Faculty of Dentistry, The British University in Egypt, Al Sherouk City 11837, Cairo, Egypt; sara.fikry@bue.edu.eg

**Keywords:** simvastatin, PRF, stem cells, bone regeneration, dental-implants

## Abstract

**Background:** Despite extensive preclinical evidence that statins enhance osteogenesis and the widespread clinical use of platelet-rich fibrin (PRF), the clinical effectiveness of statin-incorporated PRF (SIM-PRF) in limiting peri-implant crestal bone loss remains insufficiently validated. **Objectives**: To address the mentioned gap, we integrated in vitro assays on human periodontal ligament stem cells (hPDLSCs) with a controlled clinical trial to test whether SIM-PRF reduces early and 12-month marginal bone loss versus PRF alone and PRF with bone graft. **Methods:** In vitro, cytotoxicity, migration and osteogenic differentiation were assessed, in addition to the effect on basal inflammatory markers. Clinically, 24 immediate-implant cases were randomized to receive PRF, PRF+SIM, or PRF+bone graft, with CBCT-based crestal bone change measured at 0–3, 3–6, and 6–12 months. **Results:** Flow cytometry confirmed the mesenchymal identity of the isolated hPDLSCs, which exhibited dose-dependent responses to SIM treatment. Lower SIM concentrations (0.1 μM) enhanced osteogenic differentiation, as evidenced by increased mineralization, alkaline phosphatase activity, and expression of osteogenic markers (RUNX2 and osteocalcin), while maintaining cell viability and migration. Both SIM concentrations (0.1 μM and 1 μM) significantly reduced basal pro-inflammatory cytokine expression (TNF-α and IL-6). Radiographic analysis revealed significantly reduced crestal bone loss (*p* < 0.001) in the PRF-SIM and PRF-Bone groups compared to PRF alone, particularly during early postoperative intervals (0–3 and 3–6 months). Notably, no significant difference was observed between the PRF-SIM and PRF-Bone groups (*p* > 0.05) in preserving the peri-implant bone. **Conclusions**: These findings highlight the potential of SIM-loaded PRF as an effective, biocompatible, and patient-friendly approach to enhance bone regeneration and implant success.

## 1. Introduction

Tooth loss has long been associated with progressive bone resorption, creating significant challenges for functional and aesthetic rehabilitation [1,2]. While dental implants have become the gold standard for tooth replacement, their long-term success hinges on achieving and maintaining stable osseointegration, a process that remains vulnerable to biological and mechanical complications [3]. Over the years, researchers and clinicians have sought innovative strategies to enhance bone regeneration around implants, particularly in compromised sites where natural healing is insufficient. Traditional approaches, such as autogenous bone grafts and guided bone regeneration (GBR), have demonstrated efficacy but are often limited by donor site morbidity, high costs, and technical complexity [4,5]. In response, the field has turned toward bioactive agents that can stimulate the body’s innate regenerative potential, with statins, particularly simvastatin (SIM), emerging as a surprising yet compelling candidate [6,7,8].

Initially developed for the management of hypercholesterolemia, SIM is classified as a 3-hydroxy-3-methylglutaryl coenzyme A (HMG-CoA) reductase inhibitor [9]. Beyond its lipid-lowering effects, extensive research has uncovered its remarkable osteogenic properties. In vitro studies have shown that SIM upregulates bone morphogenetic protein-2 (BMP-2), a key regulator of osteoblast differentiation, while simultaneously inhibiting osteoclast activity [10]. This dual mechanism not only enhances bone formation but also mitigates excessive resorption, a critical advantage in implant dentistry, where peri-implant bone loss is a leading cause of failure. Notably, SIM has been shown to stimulate stem cells from diverse sources, including the dental pulp, periodontal ligaments, and bone marrow, suggesting its broad applicability in oral regenerative therapies [7,11,12].

Mechanistically, SIM inhibits HMG-CoA reductase, lowering mevalonate-derived isoprenoids (FPP, GGPP), which reduces prenylation of small GTPases (e.g., RhoA). This mechanism stimulates osteogenic signaling by elevating BMP-2 expression, which subsequently triggers the Smad/Runx2 pathways and stabilizes Wnt/β-catenin. Consequently, it enhances ALP activity, supports matrix mineralization, and promotes osteoblast differentiation [13]. However, the systemic administration of statins for bone regeneration is impractical due to the high doses required and potential adverse effects [14]. Conversely, localized delivery methods like platelet-rich fibrin (PRF) have become increasingly popular. PRF, a second-generation platelet concentrate, serves as an ideal scaffold due to its autologous origin, biocompatibility, and sustained release of growth factors [15]. When loaded with SIM, PRF forms a bioactive matrix that promotes gradual elution of the drug directly at the target site, thereby maximizing therapeutic efficacy while minimizing systemic exposure [16,17]. Preclinical studies have demonstrated the success of this approach in accelerating bone healing; however, clinical validation, particularly in comparison with established materials such as deproteinized bovine bone mineral, remains limited.

This study aimed to advance the translation of SIM-PRF therapy from the laboratory to clinical settings. Using a dual-phase design, we first investigated the in vitro effects of SIM on periodontal ligament stem cells (PDLSCs), assessing its influence on osteogenic differentiation and inflammatory markers. We then transitioned to a clinical trial in which patients who underwent immediate implant placement received either SIM-PRF or traditional bone graft materials. We evaluated peri-implant bone changes using cone-beam computed tomography (CBCT).

By integrating molecular insights with clinical outcomes, this study aimed to redefine new strategies in implant dentistry. If successful, SIM-PRF could offer a safer, more affordable, and effective alternative to conventional grafts, ultimately expanding treatment accessibility and improving patient prognosis. As the demand for dental implants continues to grow globally, such innovations are essential to address the biological and economic barriers that still limit optimal care.

## 2. Results

### 2.1. In Vitro Results

#### 2.1.1. Isolation and Characterization of hPDLSCs

Flow cytometry revealed that over 95% of the isolated hPDLSCs expressed typical mesenchymal stem cell surface markers, namely CD105, CD90, and CD73. In contrast, these cells exhibited very low expression levels (<5%) of hematopoietic markers, such as CD34, CD45, and HLA-DR (Supplementary Data A Figure S1).

Furthermore, hPDLSCs demonstrated the capacity for trilineage differentiation. Osteogenic differentiation was confirmed by the presence of calcium-rich nodules stained with Alizarin Red. Alcian Blue-stained mucopolysaccharides evidenced chondrogenic differentiation, and adipogenic differentiation was indicated by Oil Red O-stained lipid droplets (Supplementary Data A Figure S2).

#### 2.1.2. Cytotoxicity Results

**Figure 1:**
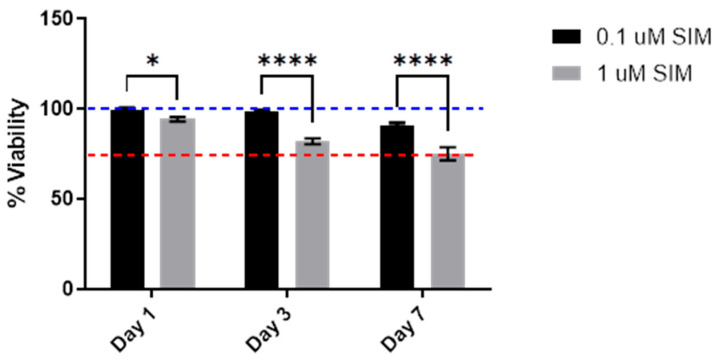
MTT assay results showing the effect of 0.1 μM and 1 μM SIM on hPDLSC viability at 1, 3, and 7 days of treatment. Cell viability was expressed as a percentage relative to that of the control. Data are represented as mean ± SD from three independent experiments, each performed in triplicate (*n* = 3). * *p* < 0.05, **** *p* < 0.0001. The blue dotted line represents the viability of untreated cells (negative control), and the red dotted line represents the threshold for cytotoxicity level (70%) compared to the control.

The MTT assay demonstrated that 1 µM SIM led to a time-dependent reduction in cell metabolic activity/viability compared to 0.1 µM SIM on days 1, 3, and 7 (*p* < 0.05). The most pronounced decrease was observed on day 7, suggesting that the lower dose did not exhibit any decreased metabolic effects (Figure 1).

#### 2.1.3. Migration Assay

**Figure 2:**
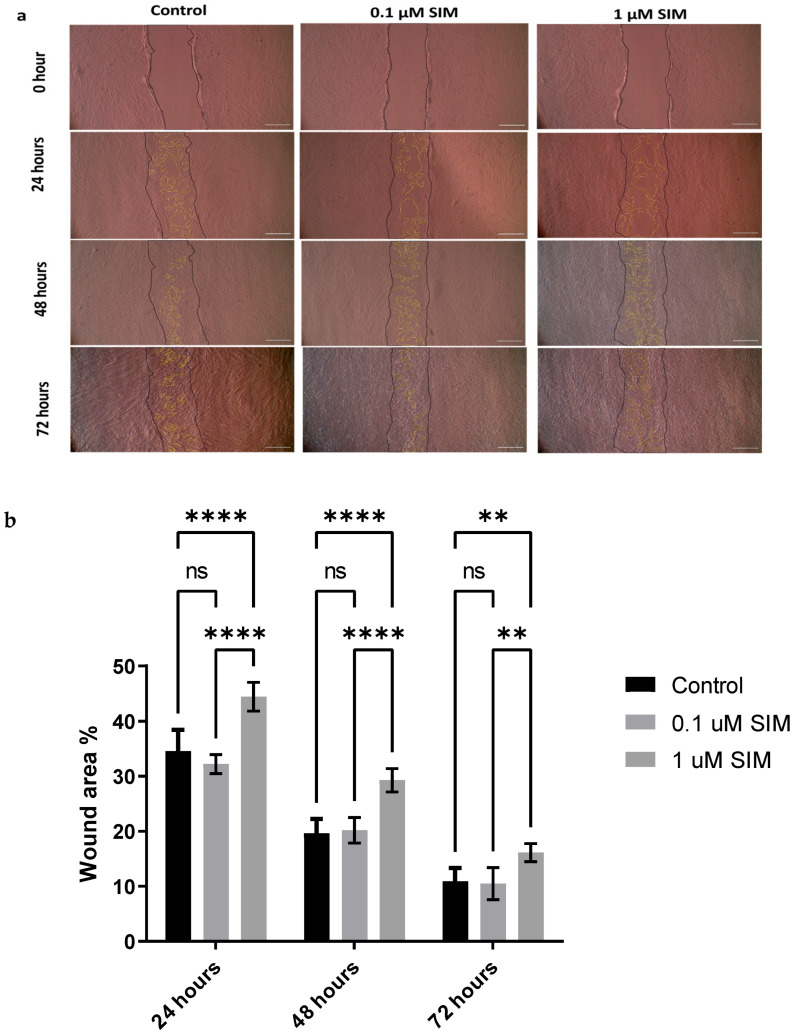
(**a**) Representative images of scratch assays at 0, 24, 48, and 72 h for control, 0.1 μM, and 1 μM SIM groups. Original wound areas from day 0 are lined by black lines, empty areas (wound areas) are lined with yellow color. Original magnification 40×, scale bar 500 μm. (**b**) Quantitative analysis of wound area. Data are presented as mean ± SD from three independent experiments, each performed in triplicate (*n* = 3). ** *p* < 0.01, **** *p* < 0.0001. ns: non-significant.

Cell migration was evaluated using a scratch assay. The 0.1 μM SIM group demonstrated a wound closure capacity similar to that of the Osteo (untreated) group. However, the 1 μM SIM group showed significantly impaired wound closure, with wider open wound areas observed at all time points (24, 48, and 72 h). Quantitative analysis confirmed that the wound area remained significantly larger in the 1 μM SIM group (*p* < 0.001), indicating a reduced migratory capacity compared to the 0.1 μM SIM and Osteo groups (Figure 2).

#### 2.1.4. Expression of Pro-Inflammatory Makers

**Figure 3:**
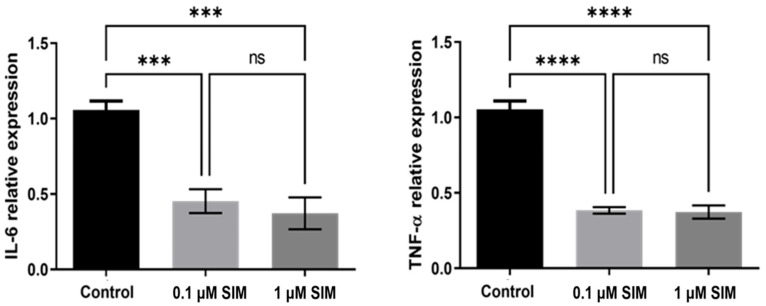
Relative expression of TNF-α and IL-6 in hPDLSCs after osteogenic induction in the control, 0.1 μM SIM, and 1 μM SIM groups. Data are shown as mean ± SD from three independent experiments, each performed in triplicate (*n* = 3). *** *p* < 0.001, **** *p* < 0.0001. ns: non-significant.

The expression levels of TNF-α and IL-6 were assessed in the different groups. Both SIM concentrations (0.1 and 1 μM) significantly reduced the basal expression of these inflammatory cytokines compared to that in the control group (*p* < 0.001). However, there was no significant difference in TNF-α (*p* = 0.9) or IL-6 (*p* = 0.5) expression between the two SIM-treated groups, suggesting a plateau effect of SIM on the modulation of the inflammatory markers’ expression (Figure 3).

#### 2.1.5. Osteogenic Differentiation

##### Alizarin Red Staining

**Figure 4:**
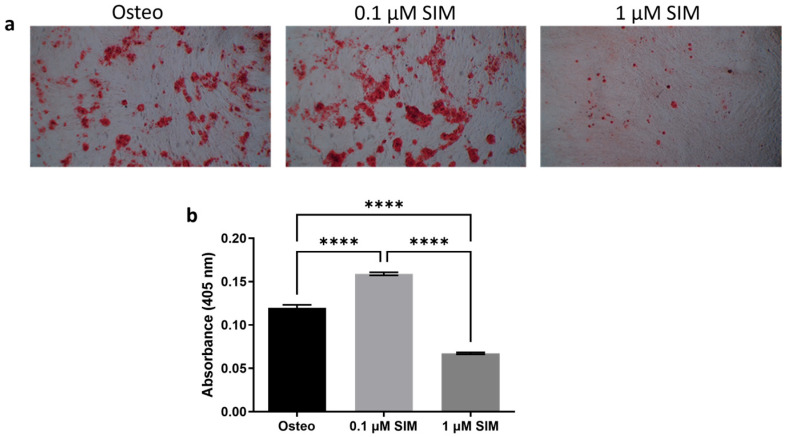
(**a**) Alizarin Red staining of mineralized nodules in the 0.1 μM SIM, 1 μM SIM, and Osteo groups. (**b**) Quantitative analysis of calcium deposition. Data are represented as mean ± SD from three experiments (*n* = 3). **** *p* < 0.0001. Original magnification ×100, scale bar 250 μm.

After 14 days of osteogenic induction, Alizarin Red staining revealed more extensive mineralized nodule formation in the 0.1 μM SIM group than in the 1 μM SIM and standard osteogenic induction (Osteo) groups. Quantitative analysis revealed significantly greater calcium deposition in the 0.1 μM SIM group (*p* < 0.0001), indicating enhanced osteogenic differentiation (Figure 4).

##### Alkaline Phosphatase (ALP) Activity

**Figure 5:**
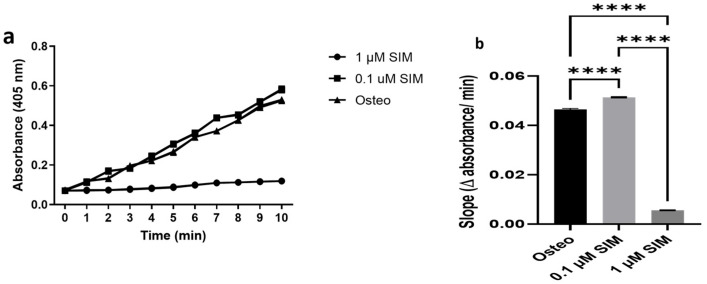
(**a**) ALP activity over time plotted as p-NP accumulation. (**b**) Statistical analysis based on the slope (initial reaction rate) of each group. The results are presented as the mean ± SD of triplicates (*n* = 3) from three experiments. **** *p* < 0.0001.

The kinetic profile of ALP activity revealed that both 0.1 μM and 1 μM SIM exhibited significantly higher ALP activity than the Osteo group (*p* < 0.0001) (Figure 5a). Comparing both SIM doses, statistical analysis of the slope, indicating the rate of dephosphorylation reaction by ALP, showed that ALP activity increased significantly using the 0.1 μM SIM dose compared with the 1 μM SIM dose (Figure 5b).

##### Expression of Osteogenic Markers

**Figure 6:**
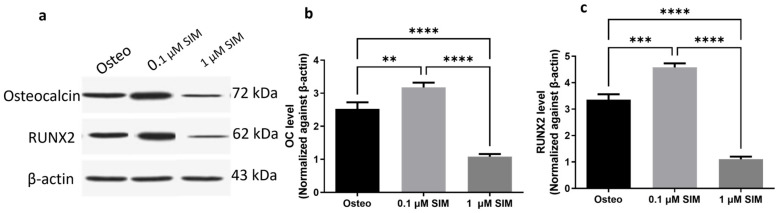
(**a**) Western blot analysis of RUNX2 and OC in hPDLSCs treated with 0.1 μM SIM, 1 μM SIM, and osteogenic medium. Densitometric analysis of (**b**) RUNX2 and (**c**) OC normalized to β-actin levels. Data are presented as mean ± SD from three repeated experiments (*n* = 3). ** *p* < 0.01, *** *p* < 0.001), **** *p* < 0.0001.

Western Blot analysis revealed that treatment with 0.1 μM SIM significantly upregulated the expression of the osteogenic proteins RUNX2 and OC compared to both the 1 μM SIM and Osteo groups (Figure 6). RUNX2 expression was significantly higher in the 0.1 μM SIM group than in the 1 μM SIM (*p* < 0.0001) and Osteo groups (*p* = 0.0002). OC expression was also significantly elevated in the 0.1 μM SIM group compared to the 1 μM SIM group (*p* < 0.0001) and the Osteo group (*p* = 0.004).

### 2.2. Clinical Results

**Figure 7:**
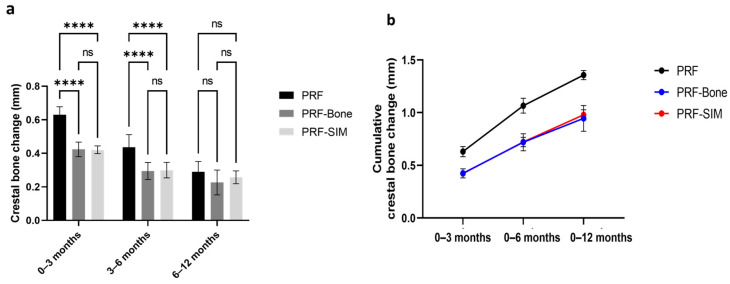
Comparison of crestal bone level changes among the PRF, PRF-Bone, and PRF-SIM groups over time. (**a**) Bar graph showing the mean crestal bone changes (in mm) at three postoperative intervals: 0–3 months, 3–6 months, and 6–12 months. Both the PRF-Bone and PRF-SIM groups demonstrated significantly reduced bone loss compared to the PRF group at 0–3 and 3–6 months intervals (*p* < 0.0001). No significant differences were observed between the PRF-Bone and PRF-SIM groups at any of the time points. A repeated-measures two-way ANOVA was performed. *n* = 8. ns: non-significant. (**b**) Cumulative crestal bone loss from baseline to 12 months. The PRF-bone and PRF-SIM groups exhibited significantly lower total crestal bone loss than the PRF group (*p* < 0.0001), with no significant difference between the two experimental groups. Data are presented as mean ± SD, *n* = 8. **** *p* < 0.0001.

**Figure 8:**
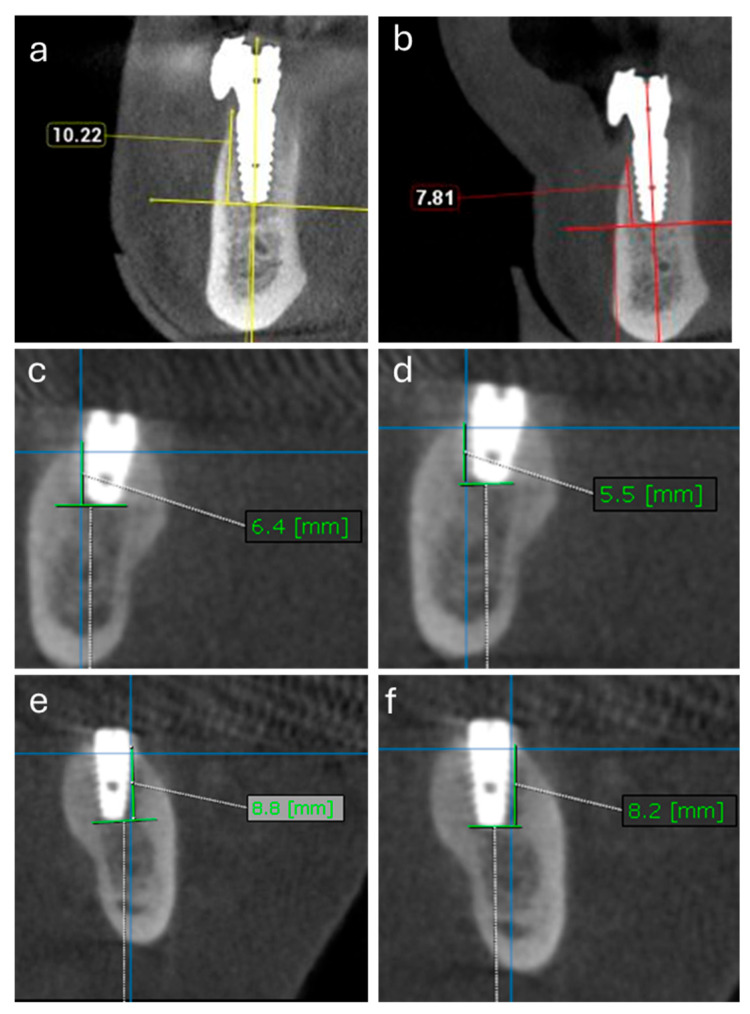
CBCT views of crestal bone levels preoperatively (**a**,**c**,**e**) and 12 months after surgery (**b**,**d**,**f**) for representative cases. PRF group: (**a**,**b**); PRF-Bone group: (**c**,**d**); PRF-SIM group: (**e**,**f**) Measurements (mm) were taken from the implant platform to the first bone–implant contact point. Scale bar 10 mm.

Statistical analysis of crestal bone level changes, the primary clinical outcome, was performed to compare the PRF, PRF-Bone, and PRF-SIM groups at three postoperative intervals: 0–3, 3–6, and 6–12 months (Figure 7 and Figure 8). At the 0–3 month interval, both the PRF-Bone and PRF-SIM groups exhibited significantly lower mean crestal bone loss than the PRF group (*p* < 0.0001), with mean changes of 0.424 ± 0.043 mm, 0.421 ± 0.023 mm, and 0.630 ± 0.048 mm, respectively. No statistically significant difference was observed between the PRF-Bone and PRF-SIM groups at this interval (*p* = 0.9).

Similar findings were recorded during the 3–6 month interval, where the PRF-Bone and PRF-SIM groups continued to show reduced crestal bone loss (0.295 ± 0.051 mm and 0.300 ± 0.047 mm, respectively) compared with the PRF group (0.436 ± 0.076 mm).

The cumulative analysis of crestal bone-level changes from baseline to 3, 6 and 12 months postoperatively indicated no significant difference between the PRF-Bone and PRF-SIM groups (*p* > 0.05). However, both groups demonstrated significantly reduced total crestal bone loss compared to that in the PRF group at the end of the designated time points.

## 3. Discussion

The present study investigated the osteogenic potential of SIM when incorporated with PRF to enhance osteogenesis and reduce peri-implant bone loss. Our findings clearly demonstrate the capacity of SIM to stimulate osteogenic differentiation and its beneficial clinical effects in preserving the crestal bone around dental implants.

The two chosen concentrations of SIM in our study were 0.1 and 1 µM due to the conflicting results, especially regarding the 1 µM concentration. Previous studies have reported that SIM concentrations of 0.05 µM, 0.1 µM, 0.5 µM, and 1 µM enhanced stem cell osteogenic differentiation [18,19]. In contrast, another study by Zhao et al. (2014) reported that SIM concentrations less than 1 µM were cytocompatible and enhanced stem cell osteogenic differentiation, while the 1 µM concentration showed significant cytotoxicity to stem cells [20].

Our results support the existing evidence highlighting the notable osteogenic properties of SIM. Previous research has demonstrated that SIM effectively increases RUNX2 expression, a crucial regulator of osteoblast differentiation, while simultaneously driving downstream expression of osteogenic markers [10,21,22]. In line with these findings, our in vitro analysis confirmed the significant upregulation of key osteogenic markers (RUNX2 and osteocalcin) when hPDLSCs were treated with SIM, reinforcing its potential as a potent promoter of bone regeneration. This dual mechanism is particularly beneficial in implant dentistry, where rapid and stable osseointegration is critical for long-term success.

Interestingly, the cellular response to SIM treatment was dose-dependent. Lower concentrations (0.1 μM) enhanced osteogenic differentiation and maintained cell viability and migratory capacity. Conversely, higher concentrations (1 μM) reduced cell viability and significantly impaired cell migration, potentially compromising the regenerative outcomes. This underscores the importance of careful dosage selection when translating SIM use from laboratory settings to clinical practice. Our findings are consistent with those of previous studies that highlighted the narrow therapeutic window of statins, beyond which they may exhibit cytotoxic effects, thereby diminishing their regenerative benefits [23,24].

Both concentrations of SIM markedly decreased the basal expression of key inflammatory cytokines in osteogenic cultures, specifically TNF-α and IL-6, despite the absence of inflammatory stimulation in the experimental conditions. Our results reflect modulation of inflammatory markers under osteogenic conditions rather than anti-inflammatory efficacy under inflammatory challenge. Nonetheless, the anti-inflammatory properties of SIM have been documented in several prior studies [6,9,10]. Excessive inflammation around dental implants can impair bone healing, hinder osseointegration, and eventually compromise implant longevity [25]. By effectively controlling inflammation, SIM-loaded PRF can potentially facilitate an improved healing environment and promote better long-term implant stability. Importantly, no significant difference was observed between the two SIM concentrations in terms of inflammation modulation, indicating a saturation effect, where increasing the dosage may not offer additional anti-inflammatory benefits.

Our clinical results show a clear advantage of SIM-loaded PRF over PRF alone in limiting early peri-implant crestal bone loss (0–3 and 3–6 months), which is the window most predictive of longer-term marginal bone level (MBL) trajectories [26]. This is important because early MBL strongly correlates with later loss, and first-year outcomes anchor classic success benchmarks (≈≤1.5 mm in year one, then ≤0.2 mm annually thereafter). By preserving bone in the early phase, SIM-PRF helps maintain implants within the accepted success envelopes and may reduce the risk of later biological complications.

The early crestal preservation we observed aligns with our cell-level findings of an increase in RUNX2/OC and maintained viability at low doses, and with prior work showing that statins upregulate BMP-2 and enhance osteoblastic activity while tempering inflammatory mediators (e.g., IL-6, TNF-α) [27,28]. Local delivery concentrates SIM at the socket, supporting osteogenesis without systemic exposure, an effect reported in extraction socket and periodontal regeneration studies using locally applied simvastatin [8]. PRF likely augments this by acting as an autologous release scaffold with its wound-healing benefits.

In our clinical study, we selected a 1.2 mg dose of SIM for local application based on prior experimental [16] and clinical studies [29,30] that investigated this dosage for local application and reported positive effects on bone regeneration. Hence, there is no correlation between the SIM doses used in our in vitro experiment with the clinically used dose.

Clinically, SIM-PRF compared to PRF combined with bone graft and membrane showed no significant difference in cumulative crestal change. In the context of dental implantology, crestal bone change of 0.5–1 mm has recently been considered clinically relevant by many clinical studies [31,32], Therefore, a crestal bone height change of 0.5 mm as observed in this study represents a clinically relevant improvement. This suggests that SIM-PRF can be a practical, cost-conscious alternative for contained or moderately compromised sockets, avoiding graft-related supply costs and donor-site morbidity associated with autogenous harvesting (such as sensory changes, pain, and dehiscence), while still achieving bone preservation comparable to more involved protocols. Selection should remain defect-specific: extensive dehiscence or combined defects may still benefit from particulate graft in addition to barrier membranes.

Our findings are consistent with those of previous studies that reported the synergistic effect of simvastatin (SIM) embedded within platelet-rich fibrin (PRF). An in vitro experiment demonstrated the time-dependent elution of SIM from A-PRF/T-PRF matrices with maintained cell viability, indicating that PRF can prolong local SIM exposure within the osteoanabolic window. The extended availability of SIM enhanced the scaffold and growth-factor effects of PRF by initiating osteogenic signaling. SIM increased BMP-2 levels and triggered Wnt/β-catenin signaling, which collectively boosted Runx2 activity, ALP expression, and mineralization. Preclinical and early clinical studies suggest that the combination of SIM and PRF is more effective for bone regeneration than using each component alone, demonstrating a synergistic advantage rather than merely additive effects. In conclusion, the osteoconductive matrix of PRF, along with its sustained-release capabilities, amplifies the osteoinductive signaling of SIM, offering a clear mechanistic rationale for the preservation of peri-implant crestal bone observed with the SIM-PRF combination [8,16,29].

Our findings align with meta-analytic evidence indicating that platelet concentrates/PRF can enhance early implant stability and, in certain contexts, mitigate marginal bone alterations [33,34]. We further suggest that augmenting PRF with local SIM may offer additional clinically significant crestal preservation without increasing surgical complexity.

SIM–PRF offers a simple, chairside, and patient-friendly way to promote bone regeneration: autologous PRF is prepared in minutes, is biocompatible with minimal immunogenic risk, releases trophic factors for early healing, and is relatively low-cost. Embedding simvastatin in PRF provides targeted and sustained local delivery at the implant site, enhancing osteogenic efficacy while avoiding high systemic doses and reducing adverse effects. The combined use of the PRF scaffold with growth factor release and SIM osteoanabolic action enhances practicality, safety, and cost-effectiveness compared to systemic statins or expensive biologics.

### Study Limitations and Directions for Future Research

This study has several limitations that should be considered when interpreting the findings. First, the sample size was small (*n* = 24), and we only tested differences among groups rather than equivalence. The follow-up was limited to 12 months, which may restrict statistical power and the ability to detect late remodeling effects. Additionally, the results may not be generalizable to females or younger patients. Second, we did not obtain histological confirmation of new bone formation; therefore, the observed radiographic changes cannot be directly equated with tissue-level regeneration. Third, we did not perform mechanical stability testing (e.g., resonance frequency analysis), which would have complemented the radiographic outcomes with functional implant stability data. Future studies with larger cohorts, longer follow-up, histomorphometric evaluation (where ethically feasible), and standardized stability metrics are warranted to validate and extend these results. Moreover, future work may include expansion of the SIM dose–response to 0.01, 0.03, 0.10, 0.30, and 1.00 µM and study the release kinetics of the drug from PRF.

## 4. Materials and Methods

### 4.1. Study Design

This integrated study combined in vitro experiments with a controlled clinical trial to evaluate the effects of SIM on stem cell osteogenesis and peri-implant bone regeneration. The research was conducted at The British University in Egypt after obtaining ethical approval (approved in March 2023, approval no. FD BUE REC 23-001) and informed consent from all participants.

### 4.2. In Vitro Experiments

#### 4.2.1. Isolation and Characterization of Stem Cells

Human periodontal ligament stem cells (hPDLSCs) were isolated from impacted third molars extracted from three healthy donors (*n* = 3). The patients were indicated for extraction at the Dental Hospital of the Faculty of Dentistry, The British University in Egypt. The study was conducted after obtaining approval from the Ethics Committee of the British University in Egypt (approval no: FD BUE 23-001) after taking informed consent from the patients. The tissues were minced and digested enzymatically using collagenase I (3 mg/mL, Sigma-Aldrich, St. Louis, MO, USA) and dispase II (4 mg/mL, Sigma) for 45 min at 37 °C. The resulting cell suspension was centrifuged, and the pellet was cultured in DMEM/F12 medium supplemented with 10% fetal bovine serum (FBS, Gibco, Grand Island, NY, USA) and antibiotics. Cells were maintained at 37 °C in a 5% CO_2_ humidified incubator until 80% confluence (passage 0, P0). Cells were subcultured and stored at −80 °C at P3, and all subsequent experiments were performed using cells from P4 [35,36,37].

#### 4.2.2. PDLSCs Characterization

To confirm the mesenchymal stem cell identity of PDLSCs, flow cytometry was performed using markers CD105, CD90, and CD73 to detect their positive presence and negative expression of CD45, CD34, and HLA-DR (Biosciences, Berkeley, CA, USA) [29,36].

Multilineage differentiation potential was tested by inducing adipogenic, chondrogenic, and osteogenic differentiation using commercial kits (R&D systems, Minneapolis, MN, USA), with Oil Red O, Alcian Blue, and Alizarin Red S staining to confirm lipid droplets, mucopolysaccharides, and mineralized nodules, respectively [38].

#### 4.2.3. SIM Treatment and Osteogenic Induction

hPDLSCs were treated with two SIM concentrations (0.1 µM and 1 µM) to assess the following:

##### Cytotoxicity

The MTT assay was used to measure cell viability after 1, 3, and 7 days of treatment. Briefly, the cells were cultured in DMEM/F12 (Gibco, Grand Island, NY, USA) for 24 h. The next day, the culture medium was replaced with a medium containing 0.1 and 1 µM SIM (Sigma) as the final concentration. At the designated time points, the culture medium was replaced by the MTT solution (1 mg/mL) and incubated for 4 h. Finally, the formed crystals were solubilized using 100 µL of DMSO, and the violet color was measured using a spectrophotometer at 570 nm. Cell viability was determined as a percentage of that of the control. Cells cultured in standard culture medium were used as the control group. The experiment was repeated three times, and each group was analyzed in triplicate (*n* = 3). The results are displayed as % viability compared to the control group (cells without treatment) using the following equation [39]:


$$\%\mathrm{Viability}=\frac{{\mathrm{Absorbance}}_{\mathrm{test}}-{\mathrm{Absorbance}}_{\mathrm{blank}}}{{\mathrm{Absorbance}}_{\mathrm{Control}}-{\mathrm{Absorbance}}_{\mathrm{blank}}}\times 100$$

##### Migration Potential

A scratch assay was used to evaluate wound closure over 72 h. Briefly, cells were cultured in DMEM/F12 in a 6-well plate until they reached 100% confluency. A scratch was induced using a sterile 200 µL pipette through the confluent layer of cells, and the cells were cultured with 0.1 and 1 µM SIM. The scratch in each well was imaged at 0, 24, 48, and 72 h using an inverted microscope. The wound area was calculated using ImageJ software (version 1.49) by a blinded investigator. The experimental groups were performed in triplicate, and the experiment was repeated three times [39].

##### Effect on Inflammatory Mediators

RT-qPCR was used to quantify Tumor necrosis factor-alpha (TNF-α) and interleukin-6 (IL-6) expression after 7 days of drugs application. The mRNA expression was normalized to that of GAPDH [18]. Total mRNA was extracted using the RNeasy Mini Kit (Qiagen, Hilden, Germany). The first cDNA strand was synthesized using the RevertAid First Strand cDNA Synthesis Kit. (Thermo Fisher Scientific, Waltham, MA, USA), and the amplification of the cDNA was performed using the Maxima Syber Green qPCR Master Mix (Thermo Fisher Scientific). The primers used in this experiment are listed in Table 1.

##### Osteogenic Differentiation: Osteogenic Differentiation Setup

PDLSCs were seeded in 6-well plates and the next day, switched to osteogenic differentiation medium (OsteoDiff; Miltenyi Biotec, Bergisch Gladbach, Germany) consisting of MEM-α, 10% FBS, ascorbic acid-2-phosphate (2.5 mg/L; Sigma-Aldrich), dexamethasone (0.1 µM; Sigma-Aldrich), and β-glycerophosphate (10 mM; Merck, Darmstadt, Germany). Simvastatin (SIM) was added at concentrations of 0.1 µM or 1 µM for 14 days. The Osteo group received osteogenic medium without SIM. All media were refreshed twice a week. After 14 days, the following parameters were assessed: (i) matrix mineralization by Alizarin Red assay, (ii) alkaline phosphatase (ALP) activity, and (iii) osteogenic markers (OPG, RUNX2) using Western blot (Antibodies; Cell Signaling Inc., Danvers, MA, USA) [40].

##### Mineralization (Alizarin Red S) Assay

After 14 days, the cells were fixed with 70% ethanol and stained with 40 mM Alizarin Red S (Sigma-Aldrich) for 30 min. Excess stain was removed with PBS, and the mineralized nodules were examined under an inverted microscope. The bound dye was then solubilized with 10% glacial acetic acid, yielding a yellow solution, and the absorbance was measured using a spectrophotometer at 405 nm. The Osteo group served as the control group [39].

##### ALP Activity Assay

After 14 days of differentiation, the monolayers were rinsed twice with PBS and once with alkaline phosphatase buffer (ALPB). One milliliter of ALPB was added to each well, followed by an equal volume of p-nitrophenyl phosphate (p-NPP; Sigma-Aldrich) pre-equilibrated at 4 °C. Immediately, 50 µL aliquots were withdrawn each minute for 10 min and mixed 1:1 with NaOH to stop the reaction. p-Nitrophenolate formation (yellow) was quantified at 405 nm and plotted against time for each group for 10 min. The slope of each reaction was determined from the time–absorbance curve for each well, indicating the rate of ALP activity [36].

Osteogenic markers detection was performed using Western blot analysis: Western blot was performed to measure osteogenic markers, including OC and RUNX2. Total protein was extracted from cell pellets using the TM Total Protein Extraction Kit (BIO-RAD Laboratories, Inc., Hercules, CA, USA), and the analysis was performed as previously described by Mohamed et al. (2024) [40]. Briefly, a total of 20 µg of protein was resolved on a 10% sodium dodecyl sulfate polyacrylamide gel electrophoresis (SDS-PAGE) gel and then transferred to a polyvinylidene difluoride (PVDF) membrane. This transfer was performed at 25 V for 7 min using a Bio-Rad Trans-Blot Turbo apparatus. The membrane was subsequently blocked with a solution containing Tris, NaCL, bovine serum albumin (BSA) and Tween 20, followed by incubation overnight at 4 °C with primary antibodies for OC (1: 500, Millipore Inc., Darmstadt, Germany) and RUNX2 (1: 1000, Cell Signaling Inc., USA) in tris-buffered saline with Tween 20 (TBST). Afterward, the membrane was treated with a secondary antibody solution (Goat anti-human IgG-HRP, 1: 5000) (Abcam, Waltham, MA, USA) for 1 h at room temperature. Protein bands were detected on photographic film (Sigma-Aldrich). Each sample was analyzed in triplicate (*n* = 3).

#### 4.2.4. In-Vitro Experiments Statistical Analysis

The normal distribution of the data was tested using the Shapiro–Wilk test. The results were analyzed using Graph-Pad Prism v8.1.0 software (GraphPad Software, San Diego, CA, USA) using a one-way ANOVA and Tukey’s post hoc tests. Data are expressed as the mean and standard deviation (SD). Values at *p* < 0.05 were considered significant.

### 4.3. Clinical Trial

#### 4.3.1. Study Design and Patient Recruitment

This study was conducted at the clinics of the Faculty of Dentistry, British University, Egypt, in accordance with the Declaration of Helsinki and approved by the Research Ethics Committee of the Faculty of Dentistry, British University, Egypt (approved on 21st of March/2023, approval no 23-001). The clinical trial was registered at ClinicalTrials.gov and accepted on 6th of September 2023, ID number: NCT06016218.

This controlled clinical study included 24 male patients aged 45–60 years who required extraction of unrestorable teeth and immediate implant placement in the mandibular posterior region. The age of all participants in the three study groups is mentioned in Tables S1 and S2 (Supplementary Data B). Recruitment was carried out for 2 months from September to October/2023.

The inclusion criteria required patients to be medically fit, with no systemic diseases or contraindications to surgery. The exclusion criteria included systemic conditions affecting bone metabolism, radiotherapy, chemotherapy, temporomandibular joint disorders, severe parafunctional habits, smoking, and psychological instability.

#### 4.3.2. Sample Size and Group Allocation

The sample size was calculated using G*Power software (v3.1.9.2) based on a prior study (Boora et al., 2015) [41] with α = 0.05 and two-tailed testing to achieve 95% power. To account for potential dropouts, the final sample included 24 implants allocated into three equal groups each group included eight patients who received one implant (*n* = 8 per group). All interventions were delivered by the clinical primary investigator.

PRF Group: PRF added into the extraction site, before implant insertion.

PRF-Bone: PRF combined with bone powder (Botiss) and covered with a pericardium membrane, added into the extraction site before implant insertion.

PRF-SIM Group: PRF combined with 1.2 mg SIM powder added into the extraction site, before implant insertion.

Randomization was achieved using a computer-generated sequence. Allocation was concealed in sequentially numbered, opaque, sealed envelopes (SNOSEs), which were opened by a dental assistant only after the patient was prepared for the surgical procedure. While the primary investigator performed the surgeries to maintain technical uniformity, the potential for bias was mitigated by ensuring that all radiographic and clinical outcome assessments were conducted by blinded investigators who had no knowledge of the treatment assignments. All patients were followed up for bone changes at 3, 6, and 12 months after surgery. The personnel who enrolled and assigned participants to the interventions had no access to the random allocation sequence.

#### 4.3.3. Surgical Procedures

Following atraumatic extraction, PRF was prepared by centrifuging 5 mL of venous blood at 3000 rpm for 12 min. The resulting fibrin clots were collected and prepared for application.

An atraumatic flapless technique was adopted for all extraction sites, then PRF, either alone, mixed with simvastatin, or mixed with bone powder, was added to the drilled site before implant insertion. Immediate implants (Vitronex, Elite, Italy) were then placed with an insertion torque range of 30–35 Ncm, using a flapless surgical technique according to standard protocols. Once the implants were in place, primary stability was checked using an ostell system, ensuring that only implants achieving a primary stability of 65–70 ISQ were included in the study.

#### 4.3.4. Prosthetic Procedures

All patients received a fixed prosthesis over their implants 4 months after implant insertion, according to a delayed loading protocol.

Primary alginate impressions (stock trays) were followed by closed-tray secondary impressions using medium-body silicone in custom trays. Jaw relations were registered and mounted on a semi-adjustable articulator for occlusal refinement. The definitive prosthesis was delivered 4 months post-implant in a delayed-loading protocol.

#### 4.3.5. Radiographic Evaluation

CBCT was done using same appropriate field of view for all patients 17 × 17 cm, voxel size 200 µm and (100 Kv, 50 mAs) using Planmeca^®^ Viso G7 CBCT machine (Planmeca Oy Asentajankatu 6 FIN-00880, Helsinki, Finland) and using ARA (artifact reduction algorithm) acquisition protocol. All radiation protection measures were carried out.

All images were interpreted with the Planmeca Romexis^®^ software (Version 6.3. Planmeca Oy Asentajankatu 6 FIN-00880, Helsinki, Finland). Images were viewed using Dell monitor (22′′ Full HD 1920 × 1080 display) in dimmed light room. On the multiplanar (MPR) screen, coronal, axial and sagittal views were reoriented to make the implant area in the center of image, a horizontal line was inserted parallel to most apical point of the fixture, then a vertical measured line inserted parallel to long axis of the fixture from the horizontal line to the level of the adjacent bone. Four vertical measurements (labial, lingual, mesial, and distal) were recorded and averaged to assess bone changes.

The scanning procedure was repeated for all patients to evaluate the longitudinal changes in peri-implant bone height. The same radiologist performed the scanning procedures for all patients to prevent inter-examiner differences.

All CBCT datasets were exported, de-identified, and assigned random alphanumeric codes by a researcher who was not involved in the measurements. The radiographic evaluator who performed the crestal bone-level measurements and all clinical investigators other than the primary investigator were blinded to the treatment allocation and time points, and the images were analyzed in a randomized order.

#### 4.3.6. Clinical-Study Statistical Analysis

Data are presented as the mean ± standard deviation (SD). Group differences across time were assessed using a two-way ANOVA, followed by Benferroni post hoc comparisons. Analyses and figures were generated using GraphPad Prism v7.00 (GraphPad Software, San Diego, CA, USA). *p* < 0.05 was considered statistically significant.

## 5. Conclusions and Future Perspectives

Overall, this integrated in vitro and clinical study provides preliminary evidence supporting the use of SIM-loaded PRF as a potential alternative for enhancing peri-implant bone regeneration. Its ease of application, favorable biological properties, and promising clinical outcomes position SIM-PRF as a compelling addition to current regenerative strategies in implant dentistry. As the global demand for dental implants continues to grow, innovations such as SIM-PRF therapy hold great promise for improving patient care, broadening treatment accessibility, and achieving superior long-term clinical outcomes.

## Figures and Tables

**Table 1:** Forward and reverse sequences of the primers used in this study.

Gene	Forward Sequence	Reverse Sequence
TNF-α	ATGTTGTAGCAAACCCTCAAGC	AGGACCTGGGAGTAGATGAGG
IL-6	ACTCACCTCTTCAGAACGAATTG	CCATCTTTGGAAGGTTCAGGTTG
GAPDH	GGAGCGAGATCCCTCCAAAAT	GGCTGTTGTCATACTTCTCATGG

TNF-α: Tumor necrosis factor-alpha; IL-6: interleukin-6.

## Data Availability

The original contributions presented in this study are included in the article and Supplementary Materials. Further inquiries can be directed to the corresponding author.

## Supplementary Materials

The following supporting information can be downloaded at: https://www.mdpi.com/article/10.3390/ph19030368/s1, Figure S1: Flow cytometry revealed that over 95% of the isolated hPDLSCs expressed typical mesenchymal stem cell surface markers, CD105, CD90, and CD73. In contrast, these cells exhibited very low expression levels (<5%) of hematopoietic markers, such as CD34, CD45, and HLA-DR. Figure S2: hPDLSCs demonstrated the capacity for trilineage differentiation. Osteogenic differentiation was confirmed by the presence of calcium-rich nodules stained with Alizarin Red. Alcian Blue-stained mucopolysaccharides evidenced chondrogenic differentiation, and adipogenic differentiation was indicated by Oil Red O-stained lipid droplets. Table S1: Patients age distribution in the three study groups; Table S2: Summary for the patients characteristics.
