# Supplementary material for: Simvastatin Enhances Stem Cell Osteogenesis and Reduces Peri-Implant Bone Loss: An In Vitro and a Randomized Clinical Study
Source: Pharmaceuticals (Basel). 2026 Feb 26;19(3):368. doi: 10.3390/ph19030368 (PMC13029674; doi:10.3390/ph19030368)
Supplement: Supplementary file 1 [file pharmaceuticals-19-00368-s001.zip › pharmaceuticals-4142366-supplementary.pdf]

Supplementary Data A: Stem cells characterization results

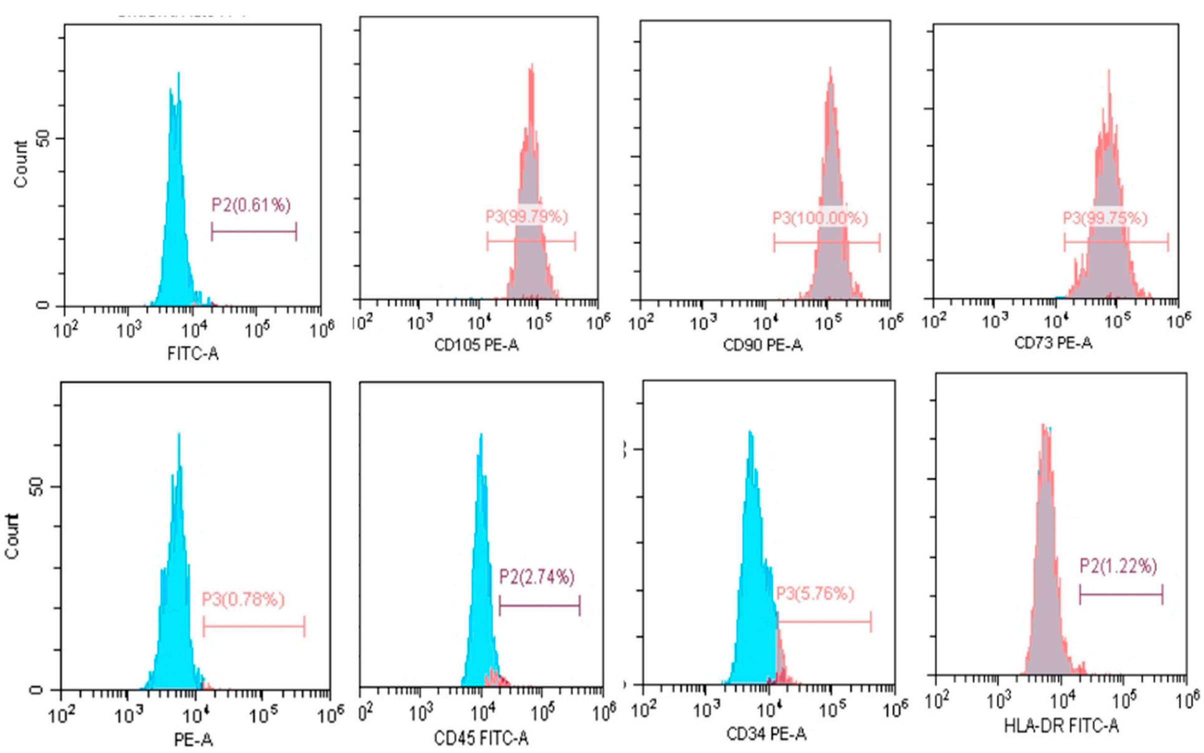

Figure S1: Flow cytometry revealed that over 95% of the isolated hPDLSCs expressed typical mesenchymal stem cell surface markers, CD105, CD90, and CD73. In contrast, these cells exhibited very low expression levels (< 5%) of hematopoietic markers, such as CD34, CD45, and HLA-DR

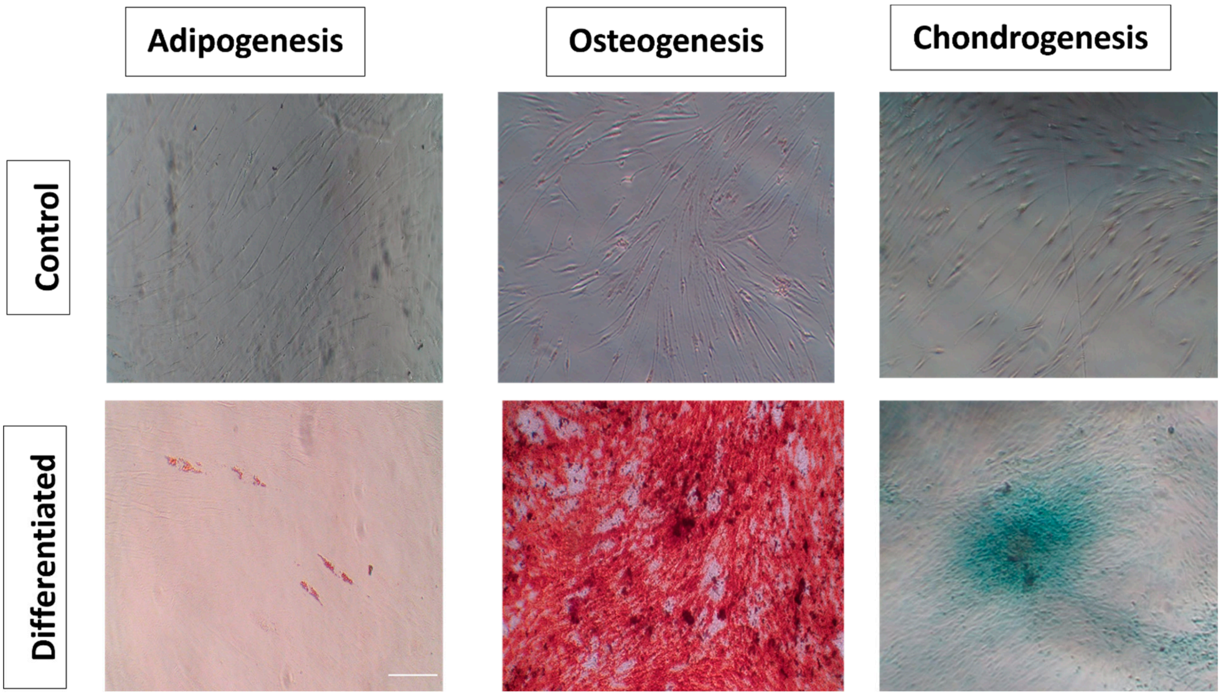

Figure S2: hPDLSCs demonstrated the capacity for trilineage differentiation. Osteogenic differentiation was confirmed by the presence of calcium-rich nodules stained with Alizarin Red. Alcian Blue-stained mucopolysaccharides evidenced chondrogenic differentiation, and adipogenic differentiation was indicated by Oil Red O-stained lipid droplets

Supplementary Data B

Table S1: Patients age distribution in the three study groups.

Patient number	Group PRF (n=8) age	Group PRF-SIM (n-8) age	Group PRF-Bone(n=8) age
Patient 1	46.0	45.4	46.0
2	47.9	47.2	48.2
3	50.1	49	50.3
4	51.3	50.9	51.4
5	53.3	52.7	53.6
6	54.5	54.6	54.7
7	56.7	56.4	56.8
8	58.6	58.2	59.0
Mean (SD)(years)	52.3 (4.1)	51.8 (4.5)	52.5 (4.1)

Table S2: Summary for the patients characteristics.

Characteristic	Group PRF (n=8)	Group PRF-SIM (n=8)	Group PRF-Bone (n=8)	Total (n=24)
**Sex:** female	0 (0%)	0 (0%)	0 (0%)	0 (0%)
Male	8 (100%)	8 (100%)	8 (100%)	24 (100%)
**Age:** ≤ 40	0 (0%)	0 (0%)	0 (0%)	0 (0%)
40-60	8 (100%)	8 (100%)	8 (100%)	24 (100%)
≥ 60	0 (0%)	0 (0%)	0 (0%)	0 (0%)
